# *Takete* and *Maluma* in Action: A Cross-Modal Relationship between Gestures and Sounds

**DOI:** 10.1371/journal.pone.0163525

**Published:** 2016-09-28

**Authors:** Kazuko Shinohara, Naoto Yamauchi, Shigeto Kawahara, Hideyuki Tanaka

**Affiliations:** 1 Division of Language and Culture Studies/Institute of Engineering, Tokyo University of Agriculture and Technology, Tokyo, Japan; 2 Cooperative Major in Advanced Health Science/Graduate School of Bio-Applications and Systems Engineering, Tokyo University of Agriculture and Technology, Tokyo, Japan; 3 Faculty of Health and Sports Science, Kokushikan University, Tokyo, Japan; 4 The Institute of Cultural and Linguistic Studies, Keio University, Tokyo, Japan; 5 Laboratory of Human Movement Science/Institute of Engineering, Tokyo University of Agriculture and Technology, Tokyo, Japan; Northeastern University, UNITED STATES

## Abstract

Despite Saussure’s famous observation that sound-meaning relationships are in principle arbitrary, we now have a substantial body of evidence that sounds themselves can have meanings, patterns often referred to as “sound symbolism”. Previous studies have found that particular sounds can be associated with particular meanings, and also with particular static visual shapes. Less well studied is the association between sounds and dynamic movements. Using a free elicitation method, the current experiment shows that several sound symbolic associations between sounds and dynamic movements exist: (1) front vowels are more likely to be associated with small movements than with large movements; (2) front vowels are more likely to be associated with angular movements than with round movements; (3) obstruents are more likely to be associated with angular movements than with round movements; (4) voiced obstruents are more likely to be associated with large movements than with small movements. All of these results are compatible with the results of the previous studies of sound symbolism using static images or meanings. Overall, the current study supports the hypothesis that particular dynamic motions can be associated with particular sounds. Building on the current results, we discuss a possible practical application of these sound symbolic associations in sports instructions.

## Introduction

### General theoretical background

One dominant theme in current linguistic theories is that sounds themselves have no meanings. This thesis—also known as the arbitrariness of the relationship between meanings and sounds—was declared by Saussure to be one of the organizing principles of natural languages [1, 2], which has had significant impacts on modern thinking about languages. In a recent review article on speech perception [3], while acknowledging some exceptions, the authors argue that “[i]n their typical function, phonetic units have no meaning” (p. 129), which shows that the arbitrariness thesis is still prevalent in the current thinking about speech perception. After all, it does not seem to be the case, at least at first glance, that, for example, /k/ itself has any inherent meanings. If there are fixed sound-meaning relationships, so the argument goes, then the same object (or the concept) should be called by the same name across all the languages (assuming that languages use the same set of sounds). This prediction is obviously false, because different languages use different strings of sounds to mean the same object/concept; e.g., the same animal is called /dɔg/ in English, /hƱnt/ in German, /$\int \text{j}\tilde{\varepsilon }$/ in French and /inu/ in Japanese, etc. The following quote from Saussure summarizes this view succinctly (pp. 67-68):

The link between signal and signification is arbitrary. Since we are treating a sign as the combination in which a signal is associated with a signification, we can express this more simply as: *the linguistic sign is arbitrary*.

(Emphasis in the original.)

…

There is no internal connexion, for example, between the idea ‘sister’ and the French sequence of sounds s-ö-r which acts as its signal. The same idea might as well be represented by any other sequence of sounds. This is demonstrated by differences between languages, and even by the existence of different languages. [2]

However, a growing body of experimental and corpus-based studies show that there is at least a stochastic tendency—or bias—for particular sounds to be associated with particular meanings—the association which is often referred to as “sound symbolism” or “sound symbolic associations” [4]. The argument for sound symbolic associations at least dates back as far as Plato’s Cratylus [5, 6]. Modern studies of sound symbolism were inspired by the pioneering work by Sapir [7], which shows that English speakers tend to associate /a/ with big images and /i/ with small images. There is now a substantial body of work showing that this size-related sound symbolism holds not only for English speakers, but also for speakers of other languages; generally, back and low vowels—those with low second formant—are associated with big images, whereas front and high vowels—those with high second formant—are associated with small images [8–17] (though cf. [18]). See also [8, 19, 20] for some classic discussions on sound symbolism, and [17] for a recent informative review, which presents a more nuanced view of non-arbitrariness in natural language.

**Fig 1:**
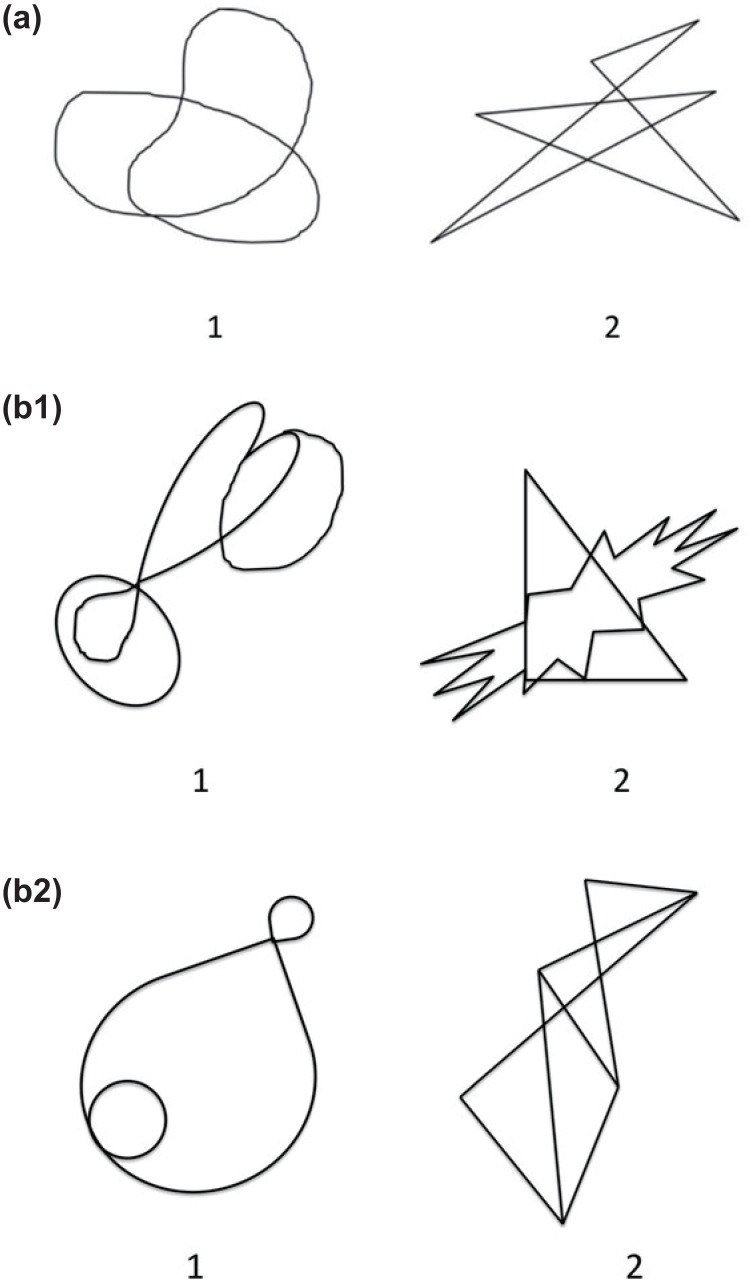
Round and angular shapes. Shapes that are associated with *maluma* and *takete*. Rounded shapes on the left tend to be associated with *maluma* and angular shapes on the right tend to be associated with *takete*. (a) Reproductions of Köhler’s original figures, (b1) shapes used in [27], and (b2) shapes used in [27].

Another well-studied case of a sound-meaning correspondence originates from the insights by Köhler [21, 22]. He pointed out that given two nonce words, *maluma* and *takete*, round shapes are more likely to be associated with the former, whereas angular shapes are associated with the latter (Fig 1(a)). These associations have been studied and replicated by a number of studies [20, 23–29] (see also [30–32] for the related “bouba-kiki” effect). A later study [27] demonstrated that this relationship is more general—the relationships hold between round shapes in general and sonorant consonants, and between angular shapes and obstruents (as those shown in Fig 1b1 and 1b2). These studies show that sounds have associations not only with linguistic meanings but also with static visual shapes.

One important emerging insight in the studies of sound symbolism is that sound symbolism is nothing but an instance of a more general cross-modal iconicity association between one perceptual domain and another [33–35]. The study by [27], for example, demonstrates that sounds can be associated not only with linguistic meanings, but also with visual shapes. Other studies have shown that particular sounds can be associated with the images of personalities [24, 36, 37], and furthermore, even shapes themselves can be associated with linguistic meanings or particular personalities (even without being mediated by sounds) [36, 37]. These results imply that a cross-modal association, of which sound symbolism is one instantiation, is a general feature of our cognition. If this hypothesis is correct, then the demonstrated examples of the sound-meaning relationships are just a tip of the iceberg.

Given this general theoretical background, one main question that is addressed in this research is as follows: If particular sounds can be associated with visual images, are such associations limited to static visual images, or can they also be associated with dynamic visual gestures like body movements? In answer to this question, we demonstrate that sounds can be associated with particular gestural motions.

Before closing the introduction, we would also like to raise one cautionary remark about what our findings—and the results of other studies of sound symbolism—would really mean to the arbitrariness thesis of Saussure [1, 2]. We are not challenging the thesis that linguistic symbols *can* be arbitrary. For example, even if the English word *big* contains a “small vowel” [I], it does not prevent the learner of this language from learning that it means “big” (though cf. [38, 39] for evidence that sound symbolism might facilitate word learning). On the other hand, we know that sound symbolic effects *do* affect the word-formation patterns in such a way that words that follow sound-symbolic patterns are more frequently found than expected by chance [15, 40]. Therefore, we do not believe that sound-symbolic mechanisms are completely outside of the linguistic system. In short, then, how the effects of sound symbolism “sneaks into” the system of arbitrary signs is an interesting issue for the cognitive science of languages (see [39] for relevant discussion). However, we do not attempt to resolve this issue in this paper.

### The current study

The current study addressed whether gestural motions can be directly associated with sounds, partly inspired by existing studies of sound symbolism in sign languages (see e.g. [41–43]). This question has been addressed by a few existing studies, which presented some video images to the participants and examined if particular motions are associated with particular sounds [29, 44] (see also [45]). Especially, the current study can be understood as an extensive follow-up study of the one conducted by Koppensteiner et al [29] with a few substantial differences. While [29] used a forced-choice paradigm, the current study used a free elicitation method, in which the participants named the given gestures rather freely. A forced choice method is amenable to a potential concern raised by Westbury [46]: “[t]he sound symbolism effects may depend largely on the experimenter pre-selecting a few stimuli that he/she recognizes as illustrating the effects of interest” (p.11). A free elicitation method deployed in the current experiment avoids this potential concern, because the sounds elicited are not pre-determined by the experimenters. (We hasten to add that we are not arguing that a forced-choice method is useless or deeply flawed in studying sound symbolic patterns. At the very least it serves to objectively confirm the intuitions that the experiments have with a large number of naive participants.)

Another aspect in which our study differs from [29] is that we used native speakers of Japanese as the target participants to address the question of how general the relationships between gestural motions and sounds are. ([29] do not report the native language of the participants. However, since the experiment was run at the University of Vienna, we conjecture that they are mainly native speakers of German.) To the extent that there is a possibility that sound symbolic patterns can partly be language-specific [14, 18, 44, 47], testing speakers of different languages is important. In addition, we also tested whether the magnitude of manual gestures can affect participants’ judgments; for example, is a large manual gesture more likely to be associated with /a/ than with /i/, a la Sapir’s [7] finding? This is a topic that was not explored by [29].

Generally, also relevant to the current study is the observation by Kunihara [48] that sound symbolism works stronger when the participants of the experiments actually pronounce the stimuli; i.e. using articulatory gestures enhances the effects of sound symbolism. This result suggests that there is a non-trivial sense in which sound symbolism is grounded in actual articulatory gestures [7, 8, 14, 26, 48–51]. For example, /a/ is considered to be large, maybe because the jaw opens the most for this vowel [52, 53]. It does not seem to be unreasonable to generalize this insight into a more general hypothesis: sounds themselves are associated with bodily gestures in general, whether they are articulatory or not (see also [41–43]). Extending on this hypothesis, at the end of the paper we address a potential practical application of this sort of research—if gestural motions have direct connections with sounds, we can make use of those associations in sports instructions [54].

## Methods

To address the question of whether some particular motions can invoke the use of particular sounds, this experiment presented carefully recorded video clips of the *maluma* and *takete* gestures to participants and asked them to name these gestures. The methodology is a free elicitation task, following the work by Berlin [26] (see also [55] for the use of similar methodology).

### Stimulus movies

#### Apparatus and setups

**Fig 2:**
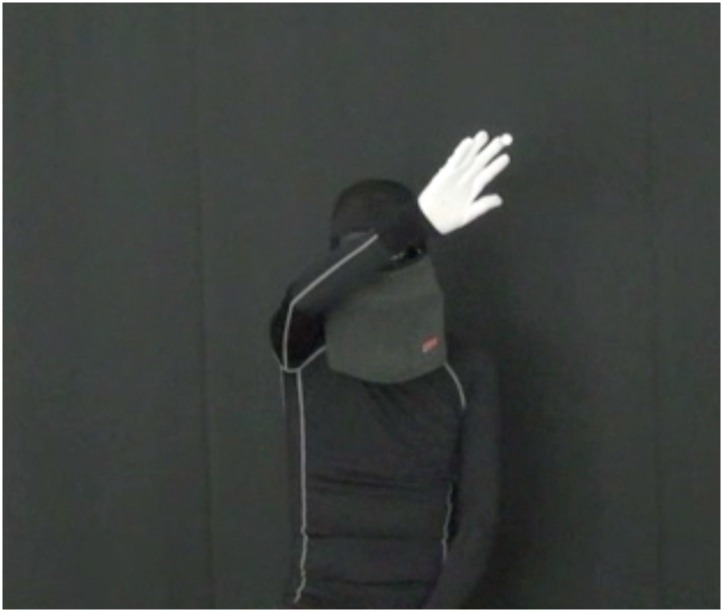
The actor. The actor who recorded the gestural stimuli.

To record the stimuli, a right-handed male served as an actor (Fig 2). The actor is the fourth author of this paper, who is able to manipulate details of his body movement very well. The actor wore a black long-sleeved shirt, a black balaclava, and a white glove on his right hand. His eyes were covered with glasses with black lenses. A spherical infrared-reflective marker (15 mm in diameter) was attached on the tip of his middle finger on the glove. In a dimly lit room, the actor sat on a high-stool in front of a black curtain and performed *maluma* and *takete* gestures with the right hand.

A digital movie camera (HDR-CX720, SONY, Japan) was placed in front of the actor; the distance between the camera and the actor was approximately 3 m. The recording covered the actor’s whole upper body, in order to capture the whole hand motions. The movie camera recorded the right hand motions with a shutter speed of 1/1000 s and a sampling rate of 60 frames per second (fps). These recording conditions were expected to provide clear cues to the movements of the white-gloved hand. See supporting information for all of the stimulus movie files that were used in the experiment.

Three high-speed cameras (OptiTrack Prime13, NaturalPoint, USA) recorded all movements of the reflective marker at 120 fps. The three-dimensional (3D) coordinates of marker position were automatically computed using motion capture system software (Motive: Body, NaturalPoint, USA). After the spatial calibration, the position errors of computed values were no more than +/- 2.5 mm in the 3D space.

#### Recording of the movie stimuli

Köhler’s original *maluma* and *takete* drawings [21, 22] were printed on a piece of paper, and placed right above the movie camera, which helped the actor to trace them with his right hand. The actor traced the shapes of *maluma* and *takete* in one stroke. The actor tried to keep the velocity of his hand movement as constant as possible, but for the *takete* movement, the acceleration profiles necessarily changed, because of the changes in directionality of the movement.

**Fig 3:**
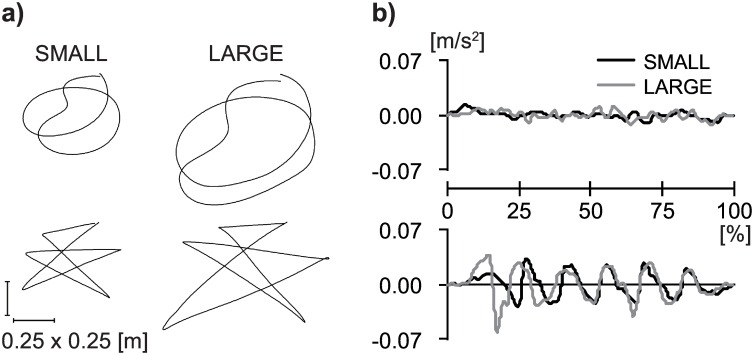
Properties of the visual stimuli. Line drawings of motion paths (a) and acceleration profiles (b) of the middle finger tip in the frontal plane for the *maluma* (the top panel) and *takete* (the bottom panel) gestures.

To examine whether the magnitude of gestures would influence their association with sounds, the actor performed each of the *maluma* and *takete* gestures in two different kinematic conditions. In the first condition (henceforth, the SMALL condition), the actor kept a motion tempo at 60 beats per minute (bpm) and completed the action within 6 s. The range of his hand movements was fit within a square range, whose length was roughly equal to his shoulder width. In the second condition (henceforth the LARGE condition), the motion tempo was 40 bpm and movement duration was 9 s. For this large condition, the actor moved his right hand approximately 1.5 times as large as the square range whose length was his shoulder width. See Fig 3a. The actor practiced these gestures until he became familiar with each condition and then repeated five recording trials for the main recording.

#### Stimulus movie selection

The 3D position data of the reflective marker were analyzed using motion analysis software (BENUS3D, Nobby-Tech, Japan) to compute five kinematic measurements on the 2D plane corresponding to the movie camera view: (1) the maximum amplitudes in the horizontal dimension (Max amp. H [m]), (2) the maximum amplitudes in the vertical dimension (Max amp. V [m]) (3) movement duration (Mov. dur. [s]), (4) mean velocity (Mean vel. [m/s]), and (5) maximum velocity (Max vel. [m/s]).

These kinematic measurements were used to choose one representative gesture motion from the five recordings for each of the two motion figures (*maluma* and *takete*) and the two kinematic conditions (SMALL and LARGE). Four gesture motions were selected according to the following criteria: (1) the movement amplitude for the LARGE condition should be 1.5 times as large as that for the SMALL condition and (2) the mean velocity and amplitude of the *takete* gesture should be similar to those of the *maluma* gesture for each kinematic condition.

#### Kinematic properties of the *maluma* and *takete* gestures

Fig 3 illustrates line drawings of motion paths and acceleration profiles of the middle finger tip on the frontal plane for the four selected gestures. The acceleration values were calculated as the second order derivative of the reflective marker position on the frontal plane against time. In the acceleration profiles, the x-axis shows the time course of the gestures in percentages; the y-axis represents the acceleration at each point in the standardized time. Fig 3a indicates that the actor reproduced hand motions that are very close to the original *maluma* and *takete* drawings of Köhler. Note however that these motion traces were not presented to our participants—they only observed the movements. Fig 3a is provided here for the sake of illustration.

Acceleration profiles were considerably flatter for the *maluma* gestures (the top panel, Fig 3b) than for the *takete* gestures (the bottom panel, Fig 3b). Fig 3b also shows that the hand was moving at an approximately constant speed in the *maluma* gestures (the top panel), reflecting smooth motion pattern. On the other hand, the *takete* gestures involve a waveform with six cycles; each cycle is a reminiscent of a sinusoid with local maximum and local minimum (the bottom panel). These waveform profiles reflect six acute changes of movement direction and movement velocity on the frontal plane in the *takete* gestures. The acceleration profiles for the SMALL and LARGE conditions are comparable, both in the *takete* and *maluma* conditions. All the kinematic measurements for the selected gestures are summarized in Table 1.

**Table 1:** Kinematic properties of the gestures used in the experiment.

Fig.	Size	Max amp.	Max amp.	Mov. dur.	Mean vel.	Max vel.
		H [m]	V [m]	[s]	[m/s]	[m/s]
*maluma*	SMALL	0.60	0.60	6.54	0.51	0.82
	LARGE	0.85	0.95	8.72	0.56	1.22
*takete*	SMALL	0.61	0.58	6.66	0.54	1.46
	LARGE	0.99	0.81	8.24	0.66	2.24

Finally, the 3D position data of the reflective marker were analyzed using motion analysis software (BENUS3D, Nobby-Tech, Japan) to produce Point-Light Display (PLD) movies of the middle finger tip. These PLD stimuli show only movements of the reflective marker, excluding any images of the actor. The motion paths and kinematic features of the PLD stimuli were identical to those of the corresponding gesture movies. While the original videos were clearly gestural movements of a human body, the PLD stimuli only involved movements of a point-light. The contrast between these two conditions was designed to address the question of whether there is a difference between human body movements and more general non-human movement patterns. (It shares the same spirit as those phonetic experiments which use non-speech stimuli for speech perception experiments [56]—see [57] for an experiment on sound symbolism using non-speech sounds).

### The elicitation task

#### Participants

Forty-four (33 male and 11 female, age 19-21) students from Tokyo University of Agriculture and Technology (TUAT) participated in this experiment. They voluntarily participated in this experiment to fulfill a requirement for course credit. All participants were native speakers of Japanese, and were naive to the purpose of the experiment. The participants had never seen Köhler’s original *maluma* and *takete* figures before participating in the experiment. The experiment was performed with the approval of the local ethics board of TUAT. The participants all signed the written informed consent form, also approved by the local ethics board of TUAT.

The participants were pseudo-randomly divided into two groups. To perform an experimental task, one group of the participants (17 male and 5 female) observed gesture movies (i.e. ACTOR group) and the other group (16 male and 6 female) observed PLD movies (i.e. PLD group).

#### Droidese word elicitation task

The task was a Droidese word invention task, originally developed by Berlin [26]. In this task, the participants were asked to name what they see in a language used by Droids (i.e. Droidese). Instead of stable drawings, as was the case for [26], our participants observed a motion movie and were asked to invent its word in Droidese. The participants were told that the sound system of Droidese includes the following consonants (/p/, /t/, /k/, /b/, /d/, /g/, /s/, /z/, /h/, /m/, /n/, /r/, /w/, and /j/) and the following vowels (/a/, /e/, /o/, /i/, and /u/). Unlike [26], /l/ was not included, because Japanese speakers do not distinguish /l/ and /r/, and /r/ is used for romanization to represent the Japanese liquid sound. /h/ was removed from the analysis following [26], because whether /h/ should be classified as an obstruent or a sonorant is debatable (e.g. [58] vs. [59]).

The participants were informed that a standard rule of Droidese phonology requires three CV syllables per word (e.g. /danizu/). They were asked to use the Japanese *katakana* orthography to write down their responses, in which one letter generally corresponds to one (C)V syllable. The *katakana* system was used because this is the orthography that is used to write previously unknown words and words spoken in non-Japanese languages (e.g. loanwords). The participants were also told that Droidese has no words with three identical CV syllables. They were also asked not to use geminates, long vowels or consonants with secondary palatalization.

With these instructions in mind, the participants were asked to invent three different names that they felt would be most appropriate for each of the four ACTOR gestures, or four PLD motions. Thus, they invented 12 different Droidese names in total.

#### Procedure

Stimulus movies were displayed on a screen in a lecture room using video player software (Quick Time Player, Apple, USA) on a PC (MacBook Air with 1.8 GHz Intel Core i7, Apple, USA). Experiments for the ACTOR and PLD groups were performed separately under the same experimental conditions.

As a practice, prior to the main trials, all of the participants observed both ACTOR movies and PLD movies that were irrelevant to the main task (e.g. pantomimic gestures of throwing and hitting). As with the main trials, they wrote down what would be appropriate words for the motions presented to them. This practice phase allowed the participants to familiarize themselves with the Droidese word invention task.

At the beginning of the test trial block, the participants observed all four stimulus movies for 30 s. Each target movie was pseudo-randomly ordered between the participants to control for any potential order effects. The participants used an answer booklet to write invented words in a designated space. Each worksheet informed the participants which of the stimulus movie (i.e. target movie) they should be observing and naming. Within each trial task, the stimulus movie was repeatedly presented to the participants, in order to assure that the participants could make up three words while observing each target movie. The test trial block took 12 minutes in total. All the participants completed the required task within the designated time limit.

#### Measurements, hypotheses and statistical analysis

Three participants in the ACTOR group and two participants in the PLD group used words that did not follow the instructions (e.g. used CVVCV words), and hence all of the data from these five participants were eliminated from the following analyses.

Following previous studies on sound symbolism, we tested the following specific hypotheses (some phonetic grounding of these hypotheses are discussed in the discussion section):

(H1) Front vowels, which involves fronting of the tongue dorsum (/i/, /e/), are more likely to be associated with the *takete* gestures than with the *maluma* gestures [8, 17, 26].
(H2) Front vowels are more likely to be associated with smaller gestures than with larger gestures [8, 10, 12, 17, 26, 60, 61].
(H3) Obstruents, which involve rise in intraoral aipressure (/p/, /t/, /k/, /s/, /b/, /d/, /g/, /z/), are more likely to be associated with angularity, whereas sonorants (/m/, /n/, /r/, /j/, /w/) are more likely to be associated with roundness [26, 27, 37].
(H4) Voiced obstruents are more likely to be associated with larger gestures than with smaller gestures, whereas voiceless obstruents are more likely to be associated with smaller gestures than with larger gestures [10, 14, 17, 26, 62].

To address these hypotheses, for each participant and each test motion, nine consonants and nine vowels in the three invented words (i.e. 3 x CVCVCV) were extracted. Then, the proportions (*P*_*ij*_) of obstruents (/p/, /t/, /k/, /b/, /d/, /g/, /s/, /z/), voiced obstruents (/b/, /d/, /g/, /z/), voiceless obstruents (/p/, /t/, /k/, /s/), and front vowels (/i/, /e/) to the total nine consonants or nine vowels were calculated. That is, we calculated the proportion of each target group of sounds to the total nine phonemes that each participant used in their three words. Further, to make these proportional values more suitable for ANOVA, we applied arcsine transformation by using the following Eq [63]:

$$X_{ij}=sin^{-1}\sqrt{P_{ij}}$$ (1)

$$P_{ij}=f_{ij}/n$$ (2)

where *f*_*ij*_ is the frequency of the target sounds produced by a participant *i* and a motion *j*, and *n* = 9. If *P*_*ij*_ is 1 or 0, they were adjusted to (*n* − 0.25)/*n* and 0.25/*n*, respectively [63].

The hypotheses were statistically assessed using three-way repeated measures ANOVA with the motion type (*maluma* vs. *takete*) and motion size (SMALL vs. LARGE) as the within-participant factors and the group (ACTOR vs. PLD) as the between-participant factor. If the three-way interaction term did not reach a significant level (*p* < 0.05), two-way repeated measures ANOVA was performed to estimate the effects of the motion type and the motion size factors. If two-way interactions of this ANOVA were significant, multiple comparison tests with the Bonferroni correction were separately performed for each combination of interests between the factor’s levels.

## Results

Three-way repeated measures ANOVA tests detected a significant group effect (ACTOR vs. PLD) only for the appearance of voiceless obstruents ($F\left(1,37\right)=6.7,p<0.05,h_{p}^{2}=0.712$): voiceless obstruents were more likely to be used for the PLD group than for the ACTOR group. No significant interactions involving the group factor were detected for any of the measurements (*p* > 0.05). Since the difference between ACTOR and PLD was negligible, we pooled the data from these two groups for the analyses and discussion that follow. The lack of difference between these two conditions implies that the patterns identified in this experiment hold for general movement patterns, and are not limited to human gestural movements.

**Fig 4:**
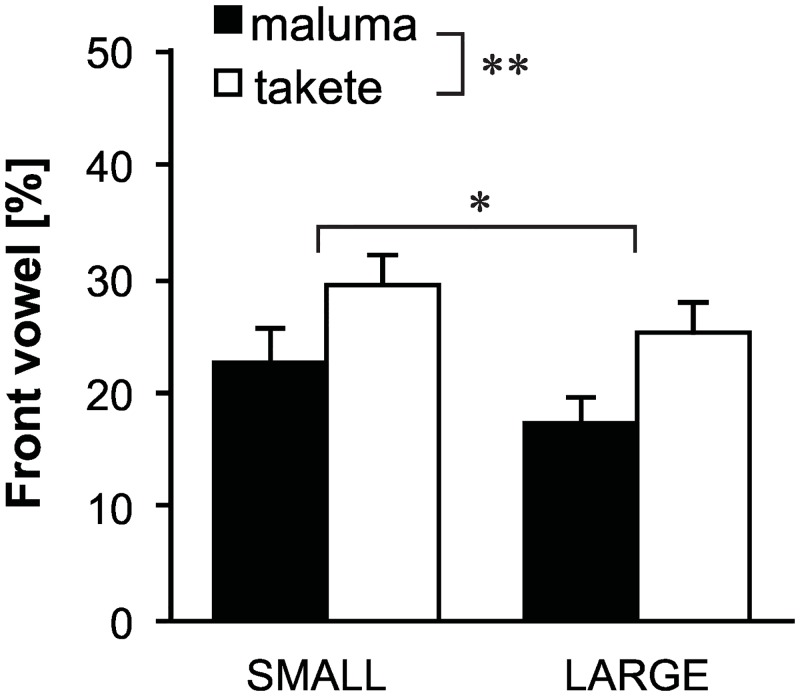
Response percentages of front vowels. Bars represents average proportions and the error bars represent standard errors. Front vowels were more likely to be associated with the *takete* motions than *maluma* motions; front vowels were also more likely to be associated with small motions than with large motions. *p < 0.05, **p < 0.01.

Fig 4 shows the average proportions (*P*_*ij*_ in Eq (2)) of front vowel responses in the elicited Droidese words. In all the result figures that follow, black bars represent words for *maluma* and white bars represent those for *takete*. The first set of bars are for the SMALL condition, and the second set of bars are for the LARGE condition. The error bars represent standard errors. The result shows that front vowels were more likely to be used for *takete* than for *maluma* ($F\left(1,38\right)=12.2,p<0.001,h_{p}^{2}=0.926$), supporting H1 formulated above. Moreover, front vowels were more likely to be used for the SMALL condition than for the LARGE condition ($F\left(1,38\right)=5.7,p<0.05,h_{p}^{2}=0.642$), supporting H2. There was no significant interaction effect ($F\left(1,38\right)=0.1,p=0.819,h_{p}^{2}=0.056$).

**Fig 5:**
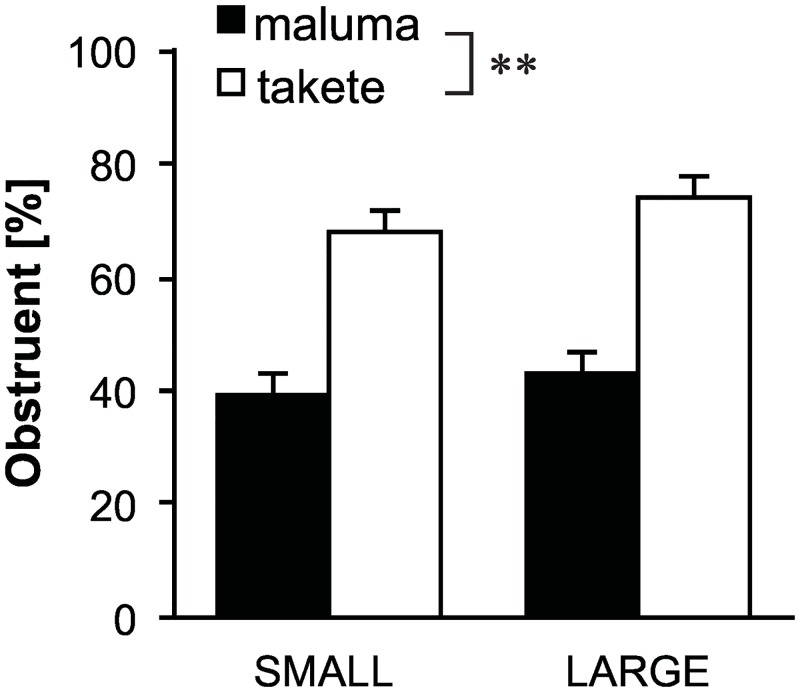
Response percentages of obstruents. Bars represents average proportions and the error bars represent standard errors. Obstruents were more likely to be associated with the *takete* motions than the *maluma* motions. *p < 0.05, **p < 0.01.

Fig 5 shows the average proportions of obstruent consonants in the elicited Droidese words. Obstruents were associated more likely with the *takete* motions than with the *maluma* motions ($F\left(1,38\right)=22.4,p<0.001,h_{p}^{2}=0.996$), which supports H3. The size of the motions did not impact the appearance of obstruents ($F\left(1,38\right)=0.3,p=0.616,h_{p}^{2}=0.078$). To the best of our knowledge, nobody has proposed a sound symbolic relationship between size and obstruency, and this lack of effect is therefore not surprising. The interaction term was not significant either ($F\left(1,38\right)=2.3,p=0.137,h_{p}^{2}=0.317$).

**Fig 6:**
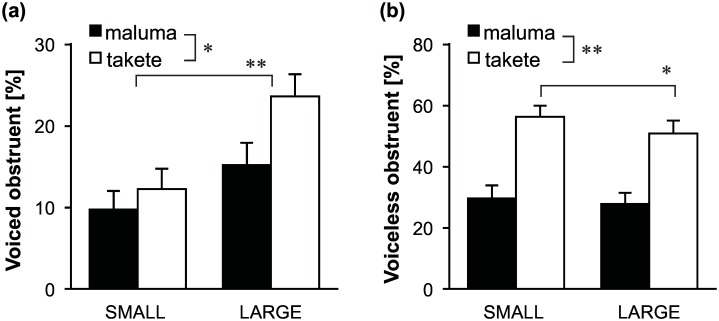
Obstruents by voicing. Bars represents average proportions and the error bars represent standard errors. Voiced obstruents were more likely to be associated with large motions. Both voiced and voiceless obstruents were more likely to be associated with the *takete* motions than the *maluma* motions. *p < 0.05, **p < 0.01.

Fig 6a and 6b illustrate the behavior of obstruents, broken down by voicing. Fig 6a shows that voiced obstruents were more likely to be associated with large motions than with small motions ($F\left(1,38\right)=16.0,p<0.001,h_{p}^{2}=0.973$), supporting H4. Both types of obstruents—voiced or voiceless—were more likely to be associated with the *takete* motions than the *maluma* motions ($F\left(1,38\right)=5.5,p<0.05,h_{p}^{2}=0.631$), supporting H3. The interaction term was not significant ($F\left(1,38\right)=2.3,p=0.141,h_{p}^{2}=0.310$). The result that voiced obstruents were more likely to be associated with the *takete* motions than with the *maluma* motions is interesting in the face of the observation that another nonce word *bouba* is often considered to represent the *maluma* picture [30]. However, *bouba* has two back vowels, which may be responsible for its association with the *maluma* picture (though cf. [32]).

Fig 6b shows that voiceless obstruents were more likely to be associated with the *takete* motions than the *maluma* motions ($F\left(1,38\right)=43.2,p<0.001,h_{p}^{2}=1.000$), which is in line with H3. There were no effects of motion sizes on the appearances of voiceless obstruents ($F\left(1,38\right)=3.7,p=0.061,h_{p}^{2}=0.468$), but there was a significant two-way interaction ($F\left(1,38\right)=4.7,p<0.05,h_{p}^{2}=0.558$). Post-hoc multiple comparison tests revealed that given the *takete* motions, voiceless obstruents appeared more often for the SMALL motions than for the LARGE motions (*p* < 0.05/4). Given the *maluma* motions, however, there were no significant differences between the SMALL and LARGE motions (*p* > 0.05/4). This complex interaction is a new finding, but at the same time we do not have a clear explanation of why voiceless obstruents were associated more with the small motions than the large motions, only for the *takete* motions.

## Discussion

### Summary

The current experiment revealed several associations between particular types of motions and particular sets of sounds: (1) front vowels are more likely to be associated with small motions than with large motions; (2) front vowels are more likely to be associated with the *takete* motions than the *maluma* motions; (3) obstruents are more likely to be associated with the *takete* motions than with the *maluma* motions; (4) voiced obstruents are more likely to be associated with large motions than small motions. Overall, the current study lends further support to the idea that dynamic motions can invoke particular sounds [29, 44, 45]. Although [29] has already shown this association, we confirmed the existence of the association using a different—and arguably better—methodology and using a set of speakers with different language background. Finding correlations between gesture sizes and some particular types of sounds—back vowels and voiced obstruents—is also new.

There was little if any difference between the ACTOR and PLD conditions. The fact that both the ACTOR condition and the PLD condition showed similar results is also interesting in that both gestural movements of a human body and non-human light movements caused similar sound symbolic patterns (cf. [64, 65]). Our participants were able to associate sounds with dynamic motions, even when the motions were movements of a point-light without any bodily gestures.

### Gestural patterns and sound symbolism

The current study has shown that back vowels are more likely to be associated with the *maluma* motions while front vowels are more likely to be associated with the *takete* motions. This finding replicates Berlin’s study who found similar sound-shape associations. The fact that the same sort of sound symbolic association holds for static visual shapes (Berlin’s study) and for dynamic movements (current study) suggests that sound symbolism is not limited to perception of static images, but also holds for the perception of dynamic motions.

The current study also demonstrated that the *takete* motions are often associated with obstruents, while sonorant sounds are often associated with the *maluma* motions. These associations again replicate the previous studies on the shape-based sound symbolism effects [26, 28, 33]. Yet again, these associations demonstrate that dynamic gestural motions can be projected onto particular sounds, expanding the scope of the traditional sound symbolism studies [29, 44].

A post-experimental questionnaire indicated that all of the participants could discriminate between the *maluma* gestures and *takete* gestures—recall, however, that no trace lines representing the motion path were presented. The current results thus raise the possibility that smooth movement patterns (for the *maluma* motions) themselves are associated with sonorant consonants and back vowels, while jagged acute movement patterns are associated with obstruents and front vowels.

### Gestural size and sound symbolism

The current study finds that back vowels are more often associated with the larger motions than the smaller motions. This finding also accords well with previous finding that back vowels are perceived to be larger [7, 10, 12, 14], arguably because the resonance cavity for the second formant is bigger for back vowels [11, 12, 14]. Yet again, this parallel suggests that dynamic motions, not just static images, can cause sound symbolic associations.

The effect of obstruent voicing on the perception of size is less well studied than the effect of vowels—however, there are a few studies suggesting that voiced obstruents are more likely to be associated with larger images than voiceless obstruents [10, 14, 17, 26, 62], and there is a reasonable articulatory basis for this association. Voicing with obstruent closure involves the expansion of the intraoral cavity due to the aerodynamic conditions imposed on voiced obstruents [66]. This articulatory movement to expand the intraoral cavity can be the source of large images.

### Further issues on sound-symbolic relationships

One issue that remains to be resolved is how direct the mapping between motions and sounds are. Do the participants directly map the actor’s gestures and PLD movements onto particular set of sounds? Or are the motion images mediated by static representations of the motion paths? The current experiment was not designed to tease apart these two possibilities, but we believe that this is an important question. If movements are directly mapped onto sounds, this connection would ultimately be related to the question of the bodily basis of sound symbolism—can sound symbolism have its roots in bodily—articulatory—movements themselves [8, 14, 30, 48]? We would like to explore this issue in more depth in future research. In order to do so, we need to examine other known cases of sound symbolism, and explore whether bodily movements can be a basis of each sound symbolic pattern.

A more general question for future research is whether it is possible that non-linguistic gestures (presented as stimuli in this experiment) are directly mapped onto articulatory gestures (cf. studies on iconicity in sign languages, e.g. [41]). We find this hypothesis to be possible, and it points to a general issue in a cross-modal perception. We often find that a cross-modal relationship holds not just between two domains, but among more than two-domains. For example, [37] finds that obstruents are associated with angular shapes and inaccessible personal characteristics, and moreover, angular shapes themselves can be associated with inaccessible personal characteristics. Given these results, an interesting question arises: how direct is the cross-modal relationship between one perceptual domain to another?

## Conclusion

Despite the fact that the relationship between meanings and sounds can be arbitrary [1], we now have a substantial body of evidence that sounds themselves have “meanings”—but what can be associated with particular sets of sounds? Most studies on sound symbolism used meanings (such as “large” or “small”), while other studies in psychology have used static visual images (like *takete* and *maluma* figures). We expanded this previous body of literature, following [29, 44], that dynamic motions can lead to sound symbolic associations. This result is compatible with the recently emerging view of sound symbolism that it is nothing but an instance of a more general cross-modal iconicity association between one perceptual domain and another [33–35].

Beyond providing further evidence for the relationship between dynamic gestures and sounds, we have yet another ultimate goal in mind. At least in Japanese, sports instructors often use onomatopoetic—sound symbolic—words to convey particular actions [54]. This practice is in accordance with the current results; both speakers and listeners know what kinds of sounds are associated with what kinds of bodily movements. Moreover, [54] shows that Japanese sports instructors use voiced obstruents more often than voiceless obstruents to express larger and stronger movements, which is compatible with the current results. [54] furthermore found that the use of long vowels and coda glottal stops is prevalent in Japanese sports instruction terms, but neither of the sound types were tested in the current experiment. In future studies, therefore, we would like to study these relationships between gestures and sounds in further detail, with the aim of inventing more effective sports instruction systems using sound symbolic words.

## Supporting Information

S1 Video Maluma and small gesture movie. (MOV)

S2 Video Maluma and large gesture movie. (MOV)

S3 Video Takete and small gesture movie. (MOV)

S4 Video Takete and large gesture movie. (MOV)

S5 Video Maluma and small PLD movie. (MOV)

S6 Video Maluma and large PLD movie. (MOV)

S7 Video Takete and small PLD movie. (MOV)

S8 Video Takete and large PLD movie. (MOV)
